# Efficacy and safety of *Centella asiatica* (L.) Urb. [Apiaceae] as a dietary supplement for glycemic control and lipid regulation in patients with type 2 diabetes: a randomized controlled trial in Thailand

**DOI:** 10.3389/fphar.2025.1680647

**Published:** 2025-11-26

**Authors:** Weeratian Tawanwongsri, Auemphon Mordmuang, Tharin Phenwan, Doungkamol Siri-archawawat

**Affiliations:** 1 Department of Internal Medicine, Division of Dermatology, School of Medicine, Walailak University, Nakhon Si Thammarat, Thailand; 2 Center of Excellence in Data Science for Health Study, Walailak University, Nakhon Si Thammarat, Thailand; 3 Department of Medical Sciences, School of Medicine, Walailak University, Nakhon Si Thammarat, Thailand; 4 School of Health Sciences, University of Dundee, Dundee, United Kingdom; 5 Department of Internal Medicine, Division of Neurology, School of Medicine, Walailak University, Nakhon Si Thammarat, Thailand

**Keywords:** *Centella asiatica* (L.) Urb. [Apiaceae], type 2 diabetes, glycemic control, lipid regulation, dietary supplement, safety

## Abstract

**Purpose:**

The objective of this study was to assess the safety and efficacy of *Centella asiatica* (L.) Urb. [Apiaceae] (CA) as a complementary dietary supplement for glycemic and lipid control in patients with type 2 diabetes mellitus.

**Patients and methods:**

A randomized, double-blind, placebo-controlled trial was conducted at Walailak University Hospital, Thailand. Participants were assigned 1:1 to receive *C. asiatica* (CA) extract 1,200 mg/day or matched placebo for 6 months. The primary outcome was glycated hemoglobin (HbA1c). Secondary outcomes were fasting plasma glucose, low-density lipoprotein cholesterol (LDL-C), and adverse events.

**Results:**

Seventy participants completed the trial (CA n = 34; placebo n = 36). Median age was 57 vs. 60 years, with women comprising 55.9% vs. 41.7% (CA vs. placebo). At 6 months, an unadjusted between-group difference in HbA1c was observed (p = 0.006); however, in the prespecified adjusted analysis (ANCOVA controlling for baseline HbA1c, diabetes duration, and sulfonylurea use) there were no between-group differences in HbA1c, fasting plasma glucose, or LDL-C. Although small within-group reductions in LDL-C were noted, the adjusted between-group comparison was not significant (p = 0.536), so lipid findings are exploratory. CA was well tolerated; mild, transient gastrointestinal symptoms were most common (5/34, 14.7%).

**Conclusion:**

CA supplementation was safe but did not produce a significant change in glycemic or lipid outcomes compared to placebo. Further studies with larger sample sizes, longer durations, and higher doses are warranted to verify potential metabolic effects.

**Clinical Trial Registration:**

This trial was registered with the Thai Clinical Trials Registry (TCTR20221219003); https://www.thaiclinicaltrials.org/show/TCTR20221219003.

## 1 Introduction

Approximately half a billion people currently live with diabetes worldwide, and this number is projected to rise by 25% by 2030 and 51% by 2045 (Saeedi et al., 2019). In Thailand, the prevalence of diabetes mellitus among the Thai population aged ≥15 years is 8.9% and continues to rise annually (Chavasit et al., 2017). Poorly controlled blood glucose in diabetes mellitus is associated with numerous serious complications. It accelerates cardiovascular disease progression, increases mortality (Wong and Sattar, 2023), and contributes to neuropathy and nephropathy, reducing quality of life (Kulkarni et al., 2024). Hyperglycemia also heightens susceptibility to infections such as *Chlamydophila pneumoniae, Haemophilus influenzae, Streptococcus pneumoniae*, severe acute respiratory syndrome coronavirus 2, influenza A, and hepatitis B by impairing immune responses, altering cellular microenvironments, and increasing oxidative stress (Chávez-Reyes et al., 2021). Additionally, chronic hyperglycemia may disrupt brain network efficiency, leading to cognitive decline (Kim et al., 2016).

Current pharmacologic treatments for diabetes mellitus regulate blood glucose through various mechanisms, including stimulating insulin secretion, inhibiting carbohydrate absorption, promoting urinary glucose excretion, and enhancing insulin sensitivity. However, these medications often present side effects such as hypoglycemia, gastrointestinal disturbances, urinary or respiratory tract infections, fluid retention, and weight gain (Alam et al., 2021; ElSayed et al., 2022). Hence, more patients are turning to botanical drugs and dietary supplements, often influenced by dissatisfaction with conventional therapies and recommendations from peers, family members, or social media (Gökkaya et al., 2023). Despite the robust scientific evidence supporting pharmacologic interventions, many patients favor botanical drugs and dietary supplements due to their perceived natural origin, cost-effectiveness, and lower incidence of side effects (Sakthiswary et al., 2014). The use of botanical drugs and dietary supplements among individuals with diabetes is substantial, with prevalence estimates ranging from 22% to 67% in the United States (Mospan, 2018). Similarly, a study in Thailand reported that 61% of patients with type 2 diabetes had been exposed to botanical drugs and dietary supplements, and 28% were actively using them at the time of the survey (Putthapiban et al., 2017). Several medicinal plants are widely recognized for their antidiabetic properties, including *Momordica charantia*, *Trigonella foenum-graecum*, *Gymnema sylvestre*, *Cinnamomum verum*, *Ocimum sanctum*, *Allium sativum*, *Panax ginseng*, *Aloe vera*, *Syzygium cumini*, and *Zingiber officinale* (Tran et al., 2020). These botanical drugs have long been used in traditional medicine to aid in blood glucose regulation and are increasingly being investigated for their potential role as complementary therapies in diabetes management. In addition to these well-established botanical drugs, *Centella asiatica* (L.) Urb. [Apiaceae] (CA) has recently emerged as a promising candidate, with preclinical studies suggesting potential antidiabetic effects.

Preclinical studies have demonstrated that CA and its triterpenes exert metabolic advantages. In nicotinamide–streptozotocin (NA–STZ) rats, CA extract 600 mg/kg for 4 weeks caused a 51% decrease in blood glucose and normalized liver weight versus the diabetic controls (Palupi et al., 2019). In a fructose–STZ animal model, CA 500–1,000 mg/kg reduced fasting glucose by approximately 40%, normalized glycolytic/gluconeogenic enzyme activities, increased muscle glycogen, and improved skeletal muscle histology (Oyenihi et al., 2019). In a high-fat diet obesity model (non-diabetic), asiatic acid 20 mg/kg caused a decrease in weight gain, fasting glucose, insulin resistance, and leptin, and enhanced lipid profile and antioxidant capacity (Rameshreddy et al., 2018). Overall, rodent studies suggest that CA has the potential to modify glucose handling, glycogen metabolism, and oxidative stress; however, human studies are limited and heterogeneous, underscoring the need for well-designed clinical trials (Sun et al., 2020).

Given the promising antidiabetic effects of CA demonstrated in preclinical models, clinical validation remains essential. Our study, therefore, aimed to evaluate the efficacy of CA in reducing blood glucose levels in patients with type 2 diabetes mellitus, along with assessing its safety profile. This investigation will contribute to the growing body of evidence supporting the use of botanical drug therapies as complementary options in diabetes management and help determine the translational potential of CA from bench to bedside.

## 2 Materials and methods

### 2.1 Study design and participants

**FIGURE 1:**
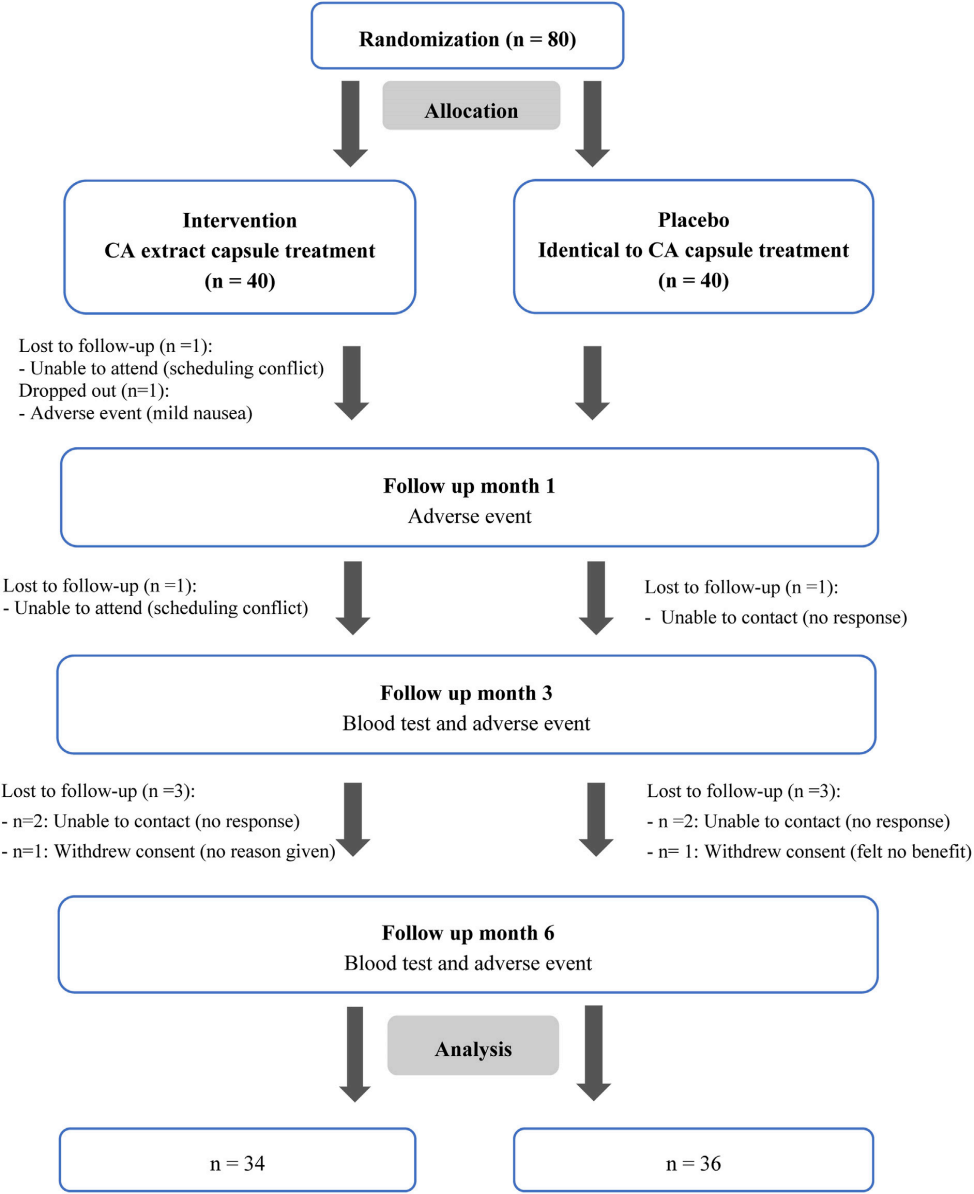
Flow diagram of participant enrollment, randomization, follow-up, and analysis in the study. CA, *Centella asiatica* (L.) Urb. [Apiaceae].

This study was a prospective, randomized, double-blind, placebo-controlled clinical trial conducted at Walailak University Hospital, Nakhon Si Thammarat, Thailand, between December 2023 and October 2024. Inclusion criteria were adults aged 18–70 years with a confirmed diagnosis of type 2 diabetes mellitus based on the 2023 Clinical Practice Guideline for Diabetes of Thailand, a stable antidiabetic regimen for at least 3 months, and willingness to provide informed consent and compliance with study procedures. Exclusion criteria included pregnancy, breastfeeding, type 1 diabetes mellitus, glycated hemoglobin (HbA1c) ≥10%, concurrent use of insulin injections, hypothyroidism or hyperthyroidism, elevated serum alanine aminotransferase (ALT) or aspartate aminotransferase (AST) levels >5 times the upper normal limit within 6 months, serum creatinine >3.5 mg/dL, glomerular filtration rate (GFR) <30 mL/min/1.73 m^2^ within 6 months, a history of severe allergy to CA, incomplete baseline data, loss to follow-up during the study period, or drug adherence <80%. Participants were randomized in a 1:1 ratio to receive either CA extract capsules or a placebo. Simple randomization was conducted using Microsoft Excel, and allocation concealment was achieved using sealed, opaque envelopes prepared by an independent investigator. Participants, treating clinicians, outcome assessors, and data analysts were blinded to group assignment. Allocation details remained concealed until the study was completed. This study was approved by the Walailak University Ethics Committee (WUEC-22–363-02). Written informed consent was obtained from all participants after a full explanation of the study procedures. The study complied with the Declaration of Helsinki and the International Conference on Harmonization of Good Clinical Practice (ICH-GCP) guidelines. This trial was registered with the Thai Clinical Trials Registry (TCTR20221219003). The participant flow is illustrated in Figure 1.

### 2.2 Compliance with ConPhyMP guidelines

This study follows the Consensus Statement on Phytochemical Characterization of Medicinal Plant Extracts (ConPhyMP) and Four Pillars of Best Practice in Ethnopharmacology. The CA formulation used (Gotu Kola Capsule) is a finished herbal medicinal product (Type A extract) that was manufactured and standardized to the Thai Herbal Pharmacopoeia. The formulation was standardized against the triterpenoid saponins, asiaticoside and madecassoside, with quality certified by the Bureau of Drug and Narcotic, Department of Medical Sciences, Thailand. The product is registered with the Thai FDA (License No. G 696/45), and the voucher specimen and production information are documented. The preparation was manufactured according to specific pharmacopeial specifications outlined in the pharmacopeial monograph, with standardized variation in the composition and in pharmaceutical quality.

Although specific analytical results (for example, HPLC chromatograms, TLC fingerprints, spectroscopic profiles, and drug-extract ratio [DER]) were not generated by the study team, but compliance with the Thai Herbal Pharmacopoeia and national regulatory approval ensures reproducibility and traceability in line with international benchmarks (Heinrich and Jalil, 2023; Heinrich et al., 2022).

### 2.3 Intervention

The CA extract capsules used in this study contained 400 mg of dried extract powder marketed under the trade name “Gotu Kola Capsule”. These capsules, registered under Thai traditional drug marketing authorization number G 696/45 and produced by the Chao Phya Abhaibhubejhr Hospital Foundation in Prachinburi Province, Thailand, contained standardized concentrations of asiaticoside (C48H78O19) and madecassoside (C48H78O20), adhering to the specifications outlined in the Thai Herbal Pharmacopoeia, with 30 μg/mL of asiaticoside and 90 μg/mL of madecassoside. The capsule product itself has undergone certification by the Bureau of Drug and Narcotic, Department of Medical Sciences, Ministry of Public Health, Thailand.

A matching placebo capsule, filled with inert pharmaceutical-grade starch powder and visually identical to the active formulation, was used to maintain blinding for both participants and study personnel. The placebo contained no active pharmacological ingredients and was manufactured in compliance with the Thai Food and Drug Administration’s Good Manufacturing Practices for food product preparation. Quality control of the placebo capsules—including verification of batch identity, safety, and microbial purity—was conducted at the Pharmaceutical Laboratory, Center for Scientific and Technological Equipment, Walailak University, Nakhon Si Thammarat, Thailand. All capsules were dispensed in sealed amber bottles labeled with anonymized randomization codes. Labeling and allocation were managed by an independent hospital pharmacist at Walailak University Hospital, who was not involved in participant care or outcome evaluation, thereby preserving the integrity of the double-blind study design. Participants in the CA group were instructed to take one capsule three times daily after meals (morning, noon, and evening), providing a total daily dose of 1,200 mg of CA extract for 6 months. The dose conforms to the manufacturer’s instructions as the standard dose and existing human-phase 1 evidence for standardized *C. asiatica* extracts demonstrating good short-term tolerability and a pharmacokinetic profile with low parent glycosides and higher metabolite exposure, which accumulated on repeat dosing (Songvut et al., 2019). Clinical evidence supports the tolerability of this dose range, which includes asiaticoside at 300 mg/day for 21 days with no serious adverse events (Paocharoen, 2010) and *C. asiatica* at 2,200 mg/day for 28 days in type 2 diabetes mellitus, where gastrointestinal adverse events did not exceed those of the placebo (Legiawati et al., 2020). Participants in the placebo group followed the same dosing schedule. All participants were advised to continue their usual medications, including antidiabetic therapies, throughout the study period. To ensure the effectiveness of blinding, both the active and placebo capsules were identical in appearance, packaging, and labeling. Both groups received self-care recommendations for patients with diabetes mellitus during follow-up visits, based on validated scripts. The content included guidance on maintaining a healthy diet, engaging in regular physical activity, adhering to prescribed medications, and recognizing and managing symptoms of hypoglycemia. Adherence was assessed by capsule count at each follow-up visit and through participant self-reports. No changes to the trial protocol were made after study initiation.

### 2.4 Outcomes and measurements

At the first screening visit, participants were asked to complete a structured interview and have a physical examination, and a blood sample was taken on the day of the study visit. Baseline characteristics such as sex, age, body mass index (BMI), blood pressure, duration of diabetes, comorbid conditions, and current medications were documented. Blood samples were collected for analysis of fasting plasma glucose, HbA1c, and serum creatinine; fasting lipids (low-density lipoproteins cholesterol (LDL-C)); and liver function tests. Predefined outcomes included HbA1c at 6 months (primary); fasting plasma glucose, LDL-C, blood pressure, and weight/BMI (secondary/exploratory efficacy); and adverse events plus renal/hepatic laboratory indices (safety), assessed at baseline, 3 months, and 6 months. Fasting plasma glucose and lipid levels (total cholesterol, HDL-C, LDL-C, and triglycerides) were assessed using enzymatic colorimetric tests as part of routine clinical laboratory tests. For HbA1c assessments, blood samples were processed using high-performance liquid chromatography (HPLC), in accordance with NGSP/DCCT reference standards. For aspartate aminotransferase (AST) and alanine aminotransferase (ALT) testing, the enzymatic method was NADH without P-5′-P, while gamma-glutamyl transferase (GGT) was measured using the para-nitrophenyl phosphate method. Direct enzymatic assays with liquid selective detergent were used for LDL-C measurements. All testing was performed in the hospital’s central lab under internal and external quality control programs. Blood pressure and anthropometric measures were taken by trained research nurses following standard operating procedures.

The primary outcomes were the changes in HbA1c levels from baseline to the end of the study. Secondary outcomes focused on fasting glucose, LDL-C, and adverse events, including the incidence of adverse drug reactions and alterations in renal and hepatic function relative to baseline. Blood tests were conducted at 3 and 6 months to monitor biochemical parameters. Clinical evaluations for adverse events were performed at months 1, 3, and 6 to ensure ongoing assessment of safety throughout the study period.

### 2.5 Sample size calculation and statistical analysis

For the primary endpoint (between-group difference in HbA1c at 6 months), the sample size was determined *a priori*. According to Nathan et al. (2009), interventions for glycemic control in patients with type 2 diabetes mellitus can lead to a reduction in HbA1c levels by approximately 0.5%–1.0%. Based on this assumption, the sample size was calculated using G*Power version 3.1.2 (Faul et al., 2009). The parameters included a mean difference of 0.5, a standard deviation of 1.0, and an effect size of 0.5. With an alpha error of 0.05, a power of 0.80, and equal allocation (1:1), the required sample size was estimated to be 34 participants per group. To account for an anticipated dropout rate of 15%, the final sample size was adjusted to 40 participants per group, resulting in a total of 80 participants for the study.

Continuous variables are summarized as means with standard deviations (SD) or medians with interquartile ranges (IQR), as appropriate; categorical variables as counts and percentages. Normality of continuous data was assessed before parametric testing. The primary analysis used baseline-adjusted ANCOVA for HbA1c at 6 months, adjusting for baseline HbA1c. Prespecified covariates were the baseline value of the outcome, diabetes duration, and sulfonylurea use. Secondary outcomes (fasting plasma glucose, LDL-cholesterol, blood pressure, weight/BMI) were analyzed with analogous baseline-adjusted models. Within-group changes (fasting blood glucose, HbA1c, GFR, AST, ALT, GGT) were explored with paired t-tests, and trajectories across baseline, 3 months, and 6 months were assessed using repeated-measures general linear models with Bonferroni correction; effect sizes are reported as Cohen’s d and partial eta squared (ηp^2^). Unadjusted between-group comparisons used independent t-tests as supportive analyses. For categorical variables, chi-square tests were applied. Analyses were complete-case (no imputation), two-sided α = 0.05, and conducted in SPSS version 18.0 (SPSS Inc., Chicago, IL).

## 3 Results

Eighty patients met the eligibility criteria, were randomized, and received study treatment. Seventy patients completed the study protocol (Figure 1). In the CA extract group, 5 patients were lost to follow-up attributable to scheduling difficulties, inability to contact, and/or withdrawal of consent. One participant discontinued due to a mild adverse event (nausea). In the placebo group, 4 patients were lost to follow-up due to inability to contact and/or withdrawal of consent. Thus, thirty-four patients in the CA extract group and thirty-six patients in the placebo group were included in the final analysis.

The baseline characteristics of both groups were comparable. The CA group comprised 55.9% female participants, while the placebo group comprised 41.7%. The median age was 57 years (IQR 38) in the CA group and 60 years (IQR 29) in the placebo group. Except for the prevalence of hypertension, diabetes duration, and baseline HbA1c level, there were no statistically significant differences between the groups at baseline (Table 1). Specifically, hypertension was more prevalent in the CA group (73.5%) compared to the placebo group (50.0%). The mean diabetes duration was 5.96 ± 5.56 years in the CA group and 3.48 ± 3.98 years in the placebo group. The CA group also exhibited a significantly higher mean baseline HbA1c level compared to the placebo group (7.62 ± 0.85 and 7.10 ± 0.62, respectively; p = 0.005). However, the mean baseline fasting blood glucose level was not significantly different between the CA group (139.32 ± 28.57 mg/dL) and the placebo group (128.36 ± 26.45 mg/dL). The types of antidiabetic medications among participants included biguanides, sulfonylureas, thiazolidinediones, dipeptidyl peptidase-4 (DPP-4) inhibitors, sodium-glucose transport protein 2 (SGLT-2) inhibitors, and glucagon-like peptide-1 (GLP-1) receptor agonists. The number of each type was similar in both groups, except for sulfonylureas, which were significantly higher in the CA group (p = 0.040).

**TABLE 1:** Demographic and laboratory characteristics.

Baseline characteristics	CA extract group (n = 34)	Placebo group (n = 36)	p-value
Female gender, N (%)	19 (55.9)	15 (41.7)	0.234
Age (year), median (min-max)	57 (30–68)	60 (39–68)	0.112
Smoking, N (%)	3 (8.8)	3 (8.3)	0.942
Alcohol drinking, N (%)	5 (14.7)	4 (11.1)	0.653
Hypertension, N (%)	25 (73.5)	18 (50.0)	*0.043* ^a^
Dyslipidemia, N (%)	33 (97.1)	35 (97.2)	0.967
Cerebrovascular disease, N (%)	1 (2.9)	5 (13.9)	0.102
Duration of diabetes (year), mean ± SD	5.96 ± 5.56	3.48 ± 3.98	*0.036* ^a^
Body Mass index (kg/m^2^), mean ± SD	27.05 ± 3.94	26.27 ± 4.68	0.452
Blood glucose (mg/dL), mean ± SD	139.32 ± 28.57	128.36 ± 26.45	0.100
HbA1c (%), mean ± SD	7.62 ± 0.85	7.10 ± 0.62	*0.005* ^a^
LDL (mg/dL), mean ± SD	114.91 ± 36.10	102.11 ± 33.86	0.133
GFR (ml/min/1.73 m^2^), mean ± SD	97.97 ± 15.93	93.00 ± 14.40	0.175
AST (U/L), mean ± SD	28.30 ± 16.08	28.70 ± 12.08	0.913
ALT (U/L), mean ± SD	35.00 ± 30.72	35.17 ± 18.74	0.980
GGT (U/L), mean ± SD	83.14 ± 53.45	61.75 ± 33.67	0.493
Current antidiabetic medications Biguanides, N (%) Sulfonylureas, N (%) Thiazolidinediones, N (%) DPP-4 inhibitors, N (%) SGLT2 inhibitors, N (%) GLP-1 receptor agonist, N (%)	29 (85.29) 13 (38.24) 3 (8.82) 7 (20.59) 4 (11.76) 0 (0.00)	22 (61.11) 6 (16.67) 4 (11.11) 5 (13.89) 4 (11.11) 2 (5.56)	0.136 *0.040* ^a^ 0.632 0.535 0.610 0.163

CA, *Centella asiatica* (L.) Urb. [Apiaceae]; SD, standard deviation; HbA1c, glycated hemoglobin; LDL, low-density lipoproteins; GFR, glomerular filtration rate; AST, aspartate aminotransferase; ALT, alanine aminotransferase; GGT, gamma-glutamyl transferase; DPP-4, dipeptidyl peptidase-4; SGLT2, sodium-glucose transport protein 2; GLP-1, glucagon-like peptide-1.

^a^ Statistically significant (p < 0.05).

### 3.1 Primary outcome

In the baseline-adjusted ANCOVA (covariates: baseline HbA1c, diabetes duration, and sulfonylurea use), the between-group difference in HbA1c at 6 months was not significant (p = 0.314; Table 4). Unadjusted values and within-group changes are summarized in Tables 2, 3. At 6 months, the CA group had a higher mean HbA1c than placebo (7.48 ± 0.77 vs. 7.01 ± 0.67; p = 0.006; Table 2); however, this unadjusted difference reflected baseline imbalance rather than a treatment effect, as confirmed by the adjusted model. Within-group trajectories across baseline, 3 months, and 6 months were analyzed using repeated-measures ANOVA with Bonferroni correction (α = 0.017), and effect sizes are reported as partial η^2^ (Tables 2, 3).

**TABLE 2:** Comparison of parameters between baseline, 3 months, and 6 months in the CA extract and placebo groups (unadjusted comparisons).

Parameters	CA extract group (n = 34)		Placebo group (n = 36)		
	Mean ± SD	p-value	Mean ± SD	p-value	p-value^a^
Blood glucose (mg/dL) At baseline At 3 months At 6 months	139.32 ± 28.57 137.71 ± 29.70 137.26 ± 31.69	1.000^b^ 1.000^b^, 1.000^c^	128.36 ± 26.45 125.85 ± 21.51 128.97 ± 29.88	1.000^b^ 1.000^b^, 0.402^c^	0.064 0.374
HbA1c (%) At baseline At 3 months At 6 months	7.62 ± 0.85 7.47 ± 0.90 7.48 ± 0.77	0.557^b^ 0.615^b^, 1.000^c^	7.10 ± 0.62 7.05 ± 0.61 7.01 ± 0.67	1.000^b^ 0.554^b^, 0.807^c^	0.057 0.006*
Body Mass index (kg/m^2^) At baseline At 3 months At 6 months	27.05 ± 3.94 27.20 ± 4.07 26.83 ± 4.32	1.000^b^ 1.000^b^, 0.578^c^	26.27 ± 4.68 26.34 ± 4.40 25.99 ± 4.68	1.000^b^ 0.492^b^, 0.280^c^	0.411 0.520
LDL (mg/dL) At baseline At 3 months At 6 months	114.91 ± 36.10 93.32 ± 25.76 95.59 ± 26.17	0.002*^b^ 0.005*^b^ *,* 1.000^c^	102.11 ± 33.86 100.81 ± 35.28 96.65 ± 30.52	1.000^b^ 1.000^b^, 0.381^c^	0.103 0.842
GFR (ml/min/1.73 m^2^) At baseline At 3 months At 6 months	97.97 ± 15.93 98.03 ± 14.52 98.16 ± 13.97	1.000^b^ 1.000^b^, 1.000^c^	93.00 ± 14.40 94.57 ± 13.24 93.56 ± 13.89	0.303^b^ 1.000^b^, 0.571^c^	0.446 0.220
AST (U/L) At baseline At 3 months At 6 months	28.30 ± 16.08 27.47 ± 13.00 28.47 ± 13.39	1.000^b^ 1.000^b^, 1.000^c^	28.70 ± 12.08 30.80 ± 9.64 31.79 ± 10.71	1.000^b^ 1.000^b^, 1.000^c^	0.298 0.232
ALT (U/L) At baseline At 3 months At 6 months	35.00 ± 30.72 33.44 ± 23.55 34.12 ± 22.00	1.000^b^ 1.000^b^, 1.000^c^	35.17 ± 18.74 38.20 ± 17.90 38.38 ± 19.96	1.000^b^ 1.000^b^, 1.000^c^	0.359 0.235
GGT (U/L) At baseline At 3 months At 6 months	83.14 ± 53.45 50.62 ± 41.36 58.79 ± 47.32	0.849^b^ 1.000^b^, 1.000^c^	61.75 ± 33.67 36.89 ± 14.97 76.88 ± 19.85	1.000^b^ 1.000^b^, 1.000^c^	0.587 0.669

^a^ p-value compared to treatment group.

^b^ p-value compared to baseline.

^c^ p-value compared 6 months–3 months.

CA, *Centella asiatica* (L.) Urb. [Apiaceae]; SD, standard deviation; HbA1c, glycated hemoglobin; LDL, low-density lipoproteins; GFR, glomerular filtration rate; AST, aspartate aminotransferase; ALT, alanine aminotransferase; GGT, gamma-glutamyl transferase.

*Statistical significance was defined as Bonferroni-adjusted p < 0.017 (0.05/3).

**TABLE 3:** Mean change in parameters from baseline to 6 months in the CA extract and placebo groups (unadjusted comparisons).

Parameters	Mean reduction from baseline to 6 months			
	CA extract group (n = 34)	Placebo group (n = 36)	p-value	95% CI
Blood glucose (mg/dL)	2.06 ± 29.60	−0.61 ± 33.11	0.528	−19.75 to 10.22
HbA1c (%)	0.14 ± 0.62	0.13 ± 0.60	0.887	−0.63 to 0.55
Body Mass index (kg/m^2^)	0.22 ± 1.19	0.27 ± 1.51	0.986	−0.65 to 0.66
LDL (mg/dL)	19.88 ± 34.31	7.00 ± 39.93	0.366	−28.61 to 10.68
GFR (ml/min/1.73 m^2^)	−0.66 ± 4.73	−0.71 ± 4.37	0.573	−2.85 to 1.59
AST (U/L)	−0.46 ± 9.97	0.20 ± 13.21	0.725	−5.34 to 7.63
ALT (U/L)	0.55 ± 19.67	2.75 ± 16.96	0.650	−8.31 to 13.20
GGT (U/L)	8.29 ± 36.50	−9.33 ± 22.60	0.469	−71.04 to 35.80

CA, *Centella asiatica* (L.) Urb. [Apiaceae]; SD, standard deviation; HbA1c, glycated hemoglobin; LDL, low-density lipoproteins; GFR, glomerular filtration rate; AST, aspartate aminotransferase; ALT, alanine aminotransferase; GGT, gamma-glutamyl transferase; CI, confidence interval.

### 3.2 Secondary outcomes

After baseline-adjusted analyses, there were no significant between-group differences at 6 months in fasting plasma glucose (p = 0.596; Table 4) or LDL-C (p = 0.536; Table 4). Although unadjusted within-group reductions were observed in the CA group at 3 and 6 months (partial η^2^ = 0.20; Tables 2, 3), the adjusted between-group contrasts were not significant. Similarly, adjusted analyses showed no between-group differences in blood pressure or weight/BMI at 6 months (Table 4); unadjusted values are reported in Tables 2, 3.

**TABLE 4:** Adjusted ANCOVA results showing mean reductions from baseline to 6 months, controlling for baseline HbA1c, duration of diabetes, and number of sulfonylureas used.

Parameters	Adjusted mean reduction from baseline to 6 months				Levene’s test of equality of error variances	
	CA extract group (n = 34)	Placebo group (n = 36)	p-value	95% CI	F	P-value
Blood glucose (mg/dL)	2.96 ± 5.73	−1.46 ± 5.56	0.596	−20.967 to 12.139	0.190	0.665
HbA1c (%)	0.10 ± 0.10	0.10 ± 0.10	0.314	−0.142 to 0.435	0.604	0.440
LDL (mg/dL)	8.90 ± 7.54	15.63 ± 7.19	0.536	−14.860 to 28.326	0.000	0.985

CA, *Centella asiatica* (L.) Urb. [Apiaceae]; HbA1c, glycated hemoglobin; LDL, low-density lipoproteins; CI, confidence interval; SD, standard deviation; ANCOVA, analysis of covariance. Values represent adjusted mean reductions from baseline to 6 months, controlling for duration of diabetes, baseline HbA1c level, and number of sulfonylureas used.

### 3.3 Safety profiles

Renal and hepatic safety outcomes are summarized in Tables 2, 3. Mean change in eGFR from baseline to 6 months was −0.66 ± 4.73 mL/min/1.73 m^2^ in the CA group and −0.71 ± 4.37 mL/min/1.73 m^2^ in the placebo group (between-group 95% CI, −2.85 to 1.59 mL/min/1.73 m^2^). For hepatic enzymes, between-group differences in mean changes from baseline to 6 months were not significant: AST −0.46 ± 9.97 vs. 0.20 ± 13.21 U/L (95% CI −5.34 to 7.63 U/L), ALT 0.55 ± 19.67 vs. 2.75 ± 16.96 U/L (95% CI −8.31 to 13.20 U/L), and GGT 8.29 ± 36.50 vs. −9.33 ± 22.60 U/L (95% CI −71.04 to 35.80 U/L).

Adverse events were collected and coded at each visit. Gastrointestinal adverse events were most common in the CA group (5/34; 14.7%), all occurring within the first 2 weeks; four cases (nausea/diarrhea) were self-limited, and one led to withdrawal. Dizziness occurred in 1/34 (2.9%). No skin reactions were observed, and no adverse events were reported in the placebo group.

## 4 Discussion

Preclinical data suggest that CA may favorably influence glucose and lipid homeostasis, but clinical evidence remains limited. In this randomized, double-blind trial, an unadjusted 6-month comparison showed higher HbA1c in the CA group than in placebo (p = 0.006); however, this reflected a higher baseline HbA1c in the CA group rather than a treatment effect. In the prespecified adjusted analysis (ANCOVA controlling for baseline HbA1c, diabetes duration, and sulfonylurea use), there were no between-group differences in HbA1c (p = 0.314), fasting plasma glucose, or LDL-C. Any small within-group LDL-C decreases should be considered exploratory.

To the best of our knowledge, there have been no clinical trials that explicitly assessed the influence of CA on glycemic control, although the mechanisms to explain its impact, such as improved insulin sensitivity, enhanced insulin secretion, and antioxidant effects, are similar to other botanicals, including curcumin, berberine, and cinnamaldehyde, which have demonstrated hypoglycemic activity and protective effects on pancreatic β-cells in both preclinical and clinical investigations (Blahova et al., 2021; Smoak et al., 2021). A recent meta-analysis of curcumin supplementation showed significant reductions in HbA1c (mean difference [MD] = −0.134%, 95% confidence interval [CI]: −0.304 to −0.037; I^2^ = 83.0) and baseline-change analysis (MD = −0.517%, 95% CI: −0.707 to −0.327; I^2^ = 61.3%) (Pathomwichaiwat et al., 2023) Similarly, it demonstrated a statistically significant reduction in fasting blood glucose (MD = −8.13 mg/dL, 95% CI: −12.18 to −4.08; I^2^ = 75.8%) with a substantial reduction in a baseline-change analysis (MD = −8.83 mg/dL, 95% CI: −13.91 to −3.76; I^2^ = 98.2%). In meta-analyses of berberine supplementation, a significant reduction in fasting plasma glucose (FPG) (weighted MD = −0.515 mmol/L, 95% CI: −0.847 to −0.183; p = 0.002; I^2^ = 89.8) has been shown (Liu et al., 2025). The cinnamon extract also produced significant reductions in blood glucose levels in individuals diagnosed with type 2 diabetes mellitus (standardized MD = −0.74, 95% CI: −1.67 to −0.18; p < 0.00001; I^2^ = 97) (Agustia et al., 2025). These studies highlight the potential for methodological overlap and common mechanism of action, although the moderate to high level of heterogeneity identified between studies does limit the generalizability of pooled results. Hence, results should be interpreted with caution, acknowledging variable quality and study design, and they should not be regarded as providing definite evidence (Imrey, 2020).

As CA supplementation at the given dosage (1,200 mg/day) did not improve glycemic parameters at 6 months versus placebo, these findings contrast with preclinical evidence showing that CA metabolites (madecassoside and asiaticoside) improve insulin secretion, β-cell function, and renal glucose regulation (Fitrianda et al., 2017; Macalalad and Gonzales, 2022; Tan et al., 2023). Several reasons for this discrepancy can be suggested. First, differences in metabolic processes among species will result in differences in pharmacokinetics for the metabolites of CA; rodents appear proficient at converting glycosides to aglycones, whilst absorption and systemic exposure for human beings are limited (Nalinratana et al., 2018; Wright et al., 2023; Wright et al., 2022). Second, the regimen of CA used in our trial (1,200 mg/day) was a dose that was considerably less extensive, on a body weight basis, than the ones employed in experimental studies (300–1,000 mg/kg) that attained a significant effect (Oyenihi et al., 2019; Palupi et al., 2019; Rameshreddy et al., 2018). Third, the patients studied had established diabetes (mean duration 4.5 years), which may have impaired the sensitivity of the β-cells and could have resulted in an attenuation of the effects required from the intervention (Verma et al., 2006; Zangeneh et al., 2006). Finally, the imprecise nature of factors such as diet, physical training, and adherence to the regimen results in additional factors that can impair the reproducibility of results, and such factors are not present under controlled laboratory conditions. All of these reflections point up the translational gap between experimental and any clinical setting, which may account for the absence of any significant metabolic effect in the present study. In addition to its glycemic effects, CA supplementation resulted in a downward trend in LDL-C levels. However, this difference was not statistically significant after adjustment. Accordingly, the lipid observations should be interpreted as exploratory. This finding is consistent with preclinical data, which suggest that CA may modify lipid metabolism and oxidative stress. Hussin et al. demonstrated in a rat model that LDL-C levels in serum were significantly decreased in CA powder-fed animals compared with controls (Hussin et al., 2009). Likewise, Zhao et al. demonstrated that a fraction of CA decreased total cholesterol and LDL-C in hyperlipidemic animals, associated with upregulation of hepatic enzymes responsible for reverse cholesterol transport (Zhao et al., 2014). It is possible, therefore, that a lipid-modifying mechanism exists that is mediated by triterpenoid metabolites such as asiatic acid and madecassic acid. Recent experimental studies have indicated that these metabolites downregulate the expression of lipogenic genes (i.e., peroxisome proliferator-activated receptor gamma, fatty acid synthase, and stearoyl-CoA desaturase 1) and inhibit metabolic inflammation from the suppression of cytokines, including TNF-α, IL-6, and MCP-1 (Chang et al., 2023; Shin et al., 2024). Although the present trial did not demonstrate a significant lipid-lowering effect, the biological plausibility observed in the experimental studies warrants further study in larger and longer clinical studies.

In this study, the incidence of adverse events in the CA group was approximately 18%, with around 15% related to gastrointestinal symptoms and 3% to dizziness. Compared to previous studies on oral CA, the safety profile observed in this study is consistent with earlier findings. Paocharoen administered 300 mg of asiaticoside daily for 21 days to patients with diabetic foot ulcers and reported no serious side effects (Paocharoen, 2010). Songvut et al. evaluated CA extract (ECa 233) in healthy Thai volunteers using single (250 mg or 500 mg) and multiple-dose (daily for 7 days) regimens; mild to moderate adverse events occurred only during the single-dose phase (Songvut et al., 2019). Similarly, Legiawati et al. used a daily dose of 2,200 mg of CA for 28 days in patients with type 2 diabetes, with gastrointestinal symptoms being the most common adverse events (15.8%–20.1%), though not significantly different from placebo (Legiawati et al., 2020). These findings collectively support the tolerability of oral CA, particularly when administered at relatively low doses and over short to moderate durations. The gastrointestinal adverse effects of CA may be attributed to several mechanisms. First, whilst CA has been effective in preventing stomach ulcers via the prevention of ethanol-induced gastric mucosal injury in rats, it is unlikely that these protective properties will apply to humans uniformly (Abdulla et al., 2010). In fact, certain individuals may experience gastrointestinal intolerance, which may be partly due to differences across individuals. Second, a triterpenoid-like metabolite of CA called asiatic acid has been shown to cause mitochondrial dysfunction in hepatocarcinoma cell lines, causing cell death. Thus, the mitochondrial uncoupling mechanism may interfere with cellular energy metabolism, causing apoptosis in gastrointestinal cells, leading to gastrointestinal disruption (Lu et al., 2016). Third, CA is capable of modulating inflammatory pathways and reducing cytokine (e.g., TNF-α, IL-6, MCP-1) secretion from interactions with TLR4, CR3, CD14, and dectin-1 receptors (Shin et al., 2024). Although these anti-inflammatory properties are mostly beneficial, these properties may affect normative gastrointestinal homeostasis and contribute to adverse reactions. Fourth, CA has been shown to reshape gut microbiota, increasing α-diversity and altering microbial community composition. Such changes may influence metabolic signaling pathways and compromise the intestinal mucosal barrier, potentially leading to gastrointestinal disturbances, including diarrhea and other digestive issues (Li et al., 2021). As prevention strategies are currently limited to managing these adverse effects, additional studies are needed to clarify the potential mechanisms underlying adverse effects and ultimately develop preventive management strategies.

We acknowledge several limitations in the study that should be considered when generalizing the findings. First, despite randomization, baseline imbalances in HbA1c levels, diabetes duration, and the higher prevalence of sulfonylurea use in the CA group may have introduced confounding bias, potentially obscuring the true effects of the intervention. Future trials should employ stratified randomization or statistical adjustments for baseline differences to minimize such bias. Second, the 6-month intervention period might not have been adequate to detect the long-term glycemic or metabolic benefits of CA supplementation. Extended follow-up durations are recommended to evaluate the sustainability of therapeutic effects and monitor for delayed outcomes. Third, despite modest within-group reductions in LDL-C, the adjusted between-group comparison was not significant; therefore, lipid outcomes should be regarded as exploratory. Other lipid parameters such as total cholesterol, HDL, and triglycerides were not measured. This limits the ability to fully assess CA’s lipid-modulating potential. Future studies should include a complete lipid profile to better define its clinical relevance. Fourth, the limited pharmacokinetic and mechanistic data available in human subjects hampers the establishment of bioavailability and systemic effects of CA’s active metabolites. Future investigations should include pharmacokinetic assessments, metabolomics, and biomarkers for β-cell function and insulin sensitivity. In this study, insulin, Homeostatic Model Assessment for Insulin Resistance (HOMA-IR), and other indices of insulin resistance were not measured, which limits the ability to corroborate CA’s potential effects on pathways influencing β-cell function and insulin resistance. Fifth, although there was adequate power in the sample size to detect a moderate effect on HbA1c, it may not have been adequate to detect smaller but clinically important differences in HbA1c and fasting glucose levels. This means that a Type II error may be present and could partially explain the negative findings for the primary outcomes of the study. There was also insufficient power to rule out potentially clinically important negative effects from CA extract. Additionally, as mentioned above, there is a need for caution in interpreting the data, especially given the wide 95% confidence intervals that indicate uncertainty regarding the meaningful clinical effects of CA extract. Sixth, the study did not perform an independent examination of the phytochemical analysis of the formulation, including the drug-to-extract ratio (DER), HPLC chromatograms, TLC fingerprints, or spectroscopic analyses. While regulatory clearance and pharmaceutical compliance indicate that acceptable quality and safety standards have been met, these factors do not fully capture the potential variability in the chemical constituents of the CA formulations. This limitation renders the interpretation of the dose–response curve more ambiguous, as the concentration of triterpenoid metabolites, such as asiaticoside and madecassoside, may differ from previous reports. Additionally, inter-study comparability is compromised due to various methodological aspects of extraction, which can result in distinct metabolite patterns and pharmacological activities. It is recommended that future studies conduct a systematic chemical analysis following the ConPhyMP modus operandi, which provides guidelines for three orthogonal fingerprinting methods and specifies the DER, enabling reproducibility and meaningful comparisons regarding pharmacological findings, dose consistency, and bioactive ingredient content. Furthermore, subsequent metabolic profile studies based on the CA formulation used and the extraction method would aid investigators in elucidating the relationship between the extracts’ chemical quality and their clinical effects. Finally, the extent of agreement among results within this study and in the clinical domain is inversely related to generalizability, as the phenomena observed are limited by the single-center nature of the data collected from a homogeneous Thai population, with individuals suffering from severe diabetes excluded from the study. Therefore, larger, multi-center studies involving diverse populations and potentially higher dosages of the CA extract are necessary to enhance statistical power, clarify clinical relevance, and improve the generalizability of the findings.

## 5 Conclusion

In this randomized, placebo-controlled trial, oral CA 1,200 mg/day was well tolerated but did not significantly improve glycemia or lipid profiles over 6 months. These findings differ from preclinical studies, which found evidence for glucose-lowering effects and antioxidant properties. These differences may be due to pharmacokinetic or pharmacodynamic differences in the standardization of the extracts used or the dosage of the active component in the human study. Future larger and longer-term trials using standardized extracts and dose-ranging are warranted to define the metabolic role of CA in type 2 diabetes.

## Data Availability

The original contributions presented in the study are included in the article/supplementary material, further inquiries can be directed to the corresponding author.
